# AI support for data scientists: An empirical study on workflow and alternative code recommendations

**DOI:** 10.1007/s10664-025-10622-4

**Published:** 2025-07-04

**Authors:** Dhivyabharathi Ramasamy, Cristina Sarasua, Abraham Bernstein

**Affiliations:** Department of Informatics, University of Zurich, Zurich, Switzerland

**Keywords:** AI support, Coding assistants, Prompt engineering, Alternative recommendations, User interfaces, Data science workflows, Computational notebooks

## Abstract

Despite the popularity of AI assistants for coding activities, there is limited empirical work on whether these coding assistants can help users complete data science tasks. Moreover, in data science programming, exploring alternative paths has been widely advocated, as such paths may lead to diverse understandings and conclusions (Gelman and Loken 2013; Kale et al. 2019). Whether existing AI-based coding assistants can support data scientists in exploring the relevant alternative paths remains unexplored. To fill this gap, we conducted a mixed-methods study to understand how data scientists solved different data science tasks with the help of an AI-based coding assistant that provides explicit alternatives as recommendations throughout the data science workflow. Specifically, we quantitatively investigated whether the users accept the code recommendations, including alternative recommendations, by the AI assistant and whether the recommendations are helpful when completing descriptive and predictive data science tasks. Through the empirical study, we also investigated if including information about the data science step (e.g., data exploration) they seek recommendations for in a prompt leads to helpful recommendations. In our study, we found that including the data science step in a prompt had a statistically significant improvement in the acceptance of recommendations, whereas the presence of alternatives did not lead to any significant differences. Our study also shows a statistically significant difference in the acceptance and usefulness of recommendations between descriptive and predictive tasks. Participants generally had positive sentiments regarding AI assistance and our proposed interface. We share further insights on the interactions that emerged during the study and the challenges that our users encountered while solving their data science tasks.

**Supplementary Information:**

The online version contains supplementary material available at 10.1007/s10664-025-10622-4.

## Introduction

In data science tasks, solutions are developed as a workflow that comprises several steps such as data preprocessing, exploration, modelling, etc. These data science solutions are generally implemented in popular computational notebook tools such as Jupyter (2015) using Python programming language (Jupyter 2015; Rule et al. 2018; Kery et al. 2017) and can contain several types of data science steps (Ramasamy et al. 2022).

Many studies advocate exploring several analytical paths that originate as a result of the choices available at a given step while implementing a data science workflow (Gelman and Loken 2013; Steegen et al. 2016) as a means to reach a reliable conclusion. However, this is not always feasible for a data scientist for several reasons, including the time and effort required (e.g., multiple projects, fast-changing data, etc.) and insufficient domain knowledge (Kale et al. 2019). Also, data scientists find it challenging to keep up to date with new techniques and methods (Kim et al. 2017). In such cases, recommending the data scientists with alternative paths could be helpful (Liu et al. 2021; Kale et al. 2019).

Recently, several studies have used machine learning approaches to generate entirely new, end-to-end data science workflows (Olson and Moore 2016; Kietz et al. 2012; Nguyen et al. 2014). Other works use rules-based techniques (Young and Holsteen 2017; Simonsohn et al. 2019; Gong et al. 2019) to generate alternative options at specific individual steps in a workflow; for example, options at the modelling step for a classification task may suggest a random forest method or an ensemble method. However, rule-based methods suffer from scalability issues. Furthermore, these studies lack user evaluation as a primary evaluation factor, which is generally the performance measured in terms of, for example, accuracy in the case of a classification task.

With the growing interest in coding assistants, mainly since the arrival of ChatGPT, it is crucial to understand how users interact with these tools in the context of data science tasks. Existing literature has predominantly focused their investigation of coding assistants in traditional programming tasks (Barke et al. 2023; Zamfirescu-Pereira et al. 2023; Ross et al. 2023; Nam et al. 2023; Liang et al. 2023). Previous investigations within the data science domain have primarily been interview studies (Wang et al. 2019; Zhang et al. 2020). In our study, to the best of our knowledge, we provide the first *empirical* investigation of how data scientists use AI-based coding assistants in the context of data science tasks and interact with the recommendations generated by these AI assistants. Specifically, we investigate whether an AI assistant can generate helpful recommendations, including alternative recommendations, for the subsequent next step in a data science workflow in order to support users in their exploration of *‘garden of forking paths’* (Gelman and Loken 2013). We assess the helpfulness of these recommendations based on their acceptability to users and whether they help the users complete the data science tasks successfully.

Additionally, as prompts can guide the model towards desired outputs (Zamfirescu-Pereira et al. 2023), we investigate whether adding the specific data science workflow step information in the prompt can lead to a higher number of recommendations accepted by the data scientists. In particular, we hypothesise that given an incomplete, user, or AI-generated workflow; an AI-based coding assistant can generate (alternative) acceptable and helpful recommendations to the user with the help of data science step information. We also investigate if and at what steps (e.g., data-preprocessing, modelling, evaluation) in a workflow alternative recommendations are more helpful in completing the data science task.

Finally, we investigate whether there are differences when it comes to solving the different types of data science tasks — descriptive and predictive — using AI assistance.

We conduct our study in a computational notebook (Jupyter) environment, given its popularity among data scientists. With this, we contribute to understanding how data scientists interact with such LLM-based AI coding assistants in notebooks, which is currently limited (McNutt et al. 2023). To conduct the study, we implemented a simple interface, ***C****ollaborative* ***A****ssistan* ***T*** *for Data* ***Sci****ence*, that supports interaction with AI assistants within computational notebooks. *CATSci* is particularly designed to support data science tasks by allowing the user to add data science workflow information (Ramasamy et al. 2022). Furthermore, through a survey, we provide qualitative insights into the expectations and challenges faced by the data scientists who participated in our study. Overall, our contributions include:

- an empirical study to understand whether the recommendations generated by an AI-based code assistant (with and without alternative solutions) are helpful to complete descriptive and predictive data science tasks
- analysis of whether adding data science workflow information in the prompt can lead to helpful recommendations for data scientists
- understanding of data scientists’ need for alternatives at different stages of a data science workflow
- insights into Human-AI interaction with coding assistants for data science tasks through a qualitative analysis
- a model-agnostic Jupyter plugin providing user interface support to interact with an AI assistant (Generative Pre-trained Transformer 4 (GPT-4) is used as the assistant in this study) for the implementation of data science tasks and its user evaluation
- a log dataset of human-AI interactions, which includes user prompts, AI-generated recommendations in response to those prompts, and the corresponding user feedback on those recommendations.

The remainder of the paper has the following structure: Section 2 discusses the relevant literature background and introduces the research questions (RQs). In Section 4, we introduce the design and implementation of the system that provides interface support in computational notebooks. In Section 5, we elaborate on the details regarding the empirical study and discuss the results in Sections 6 and 7. Section 8 expands on the results and their implications. Finally, we list the limitations with respect to the empirical study in Section 9 and conclude with Section 10.

## Background and Research Questions

While the existing literature has studied how developers interact with coding assistants in generic programming tasks (Barke et al. 2023; Zamfirescu-Pereira et al. 2023; Ross et al. 2023; Nam et al. 2023), there is a limited understanding of how effective existing AI assistants are in providing (alternative) code recommendations to solve data science tasks and how to improve them.

### LLM-based Coding Assistants

Recent advancements in large language models (LLMs) have significantly expanded the capabilities of traditional coding assistants beyond simple token-completion tasks. Several LLM-based models now power these advanced assistants, including OpenAI’s GPT-4 (Achiam et al. 2023), Meta’s Code Llama (Roziere et al. 2023), Google PaLM (Chowdhery et al. 2023), and DeepMind AlphaCode (Li et al. 2022). While these models are trained on both code and natural language data, others have emerged with a primary focus on code, such as Code Llama - Python and CodeParrot (Tunstall et al. 2022) (both specializing in Python), and PolyCoder (Xu et al. 2022) (trained for multiple programming languages). Existing literature that evaluates LLM-based models consistently highlights the GPT model as one of the top performers in code completion tasks. Although these models have been evaluated in traditional programming contexts, such as aiding students learning to code (Kazemitabaar et al. 2024, 2023), solving LeetCode problems (Coignion et al. 2024), and their performance on benchmarks (Liu et al. 2024) such as HumanEval dataset (Xu et al. 2022), their effectiveness in assisting with data science programming remains largely unexplored.

### Generating Alternatives for Data Science Tasks

Past works address the issue of generating alternatives by focusing on diversifying (Bar El et al. 2019) the options available to solve a task at a specific step. With the availability of pre-defined function calls, the most prominent way of generating alternatives is based on selecting diverse methods available through API calls. Gong et al. (2019) discuss diversity and identify different areas of diversification in machine learning. In their work within the context of the data exploration step, Bar El et al. (2019) use diversity as a factor to generate different views of a dataset. Merrill et al. (2021) created alternatives for data science code snippets using diffs. In the area of statistical analysis, Gong et al. (2019); Simonsohn et al. (2019); Young and Holsteen (2017) address the need for diverse analysis by creating task-specific tools that use a rules-based approach to generate multiple analyses. However, this approach works well only for simple statistical tasks. For example, Young and Holsteen (2017) developed a STATA module that uses simple variable substitution for multi-model analysis. In our work, we leverage large language models to generate the alternatives.

### Evaluating Alternatives in Data Science Tasks

Given that data science development processes involve several implicit choices that are subjective (Gelman and Loken 2013; Ramasamy et al. 2023), recent studies recommend performing multiverse analysis (Steegen et al. 2016; Dragicevic et al. 2019) before arriving at a conclusion. Multiverse analysis in data science is the practice of performing and evaluating all possible and reasonable paths while solving the task before arriving at a conclusion (Steegen et al. 2016). In John Tukey’s words, “it is right that each of us try many things that do not work — that we tackle more problems than we make expert analyses of” (Tukey et al. 1977).

However, the utility of an alternative relies on the user’s ‘motivations and constraints’ (Kale et al. 2019). Therefore, to evaluate whether the alternatives generated by an existing assistant are helpful, we conduct an empirical study and investigate the following research question: 
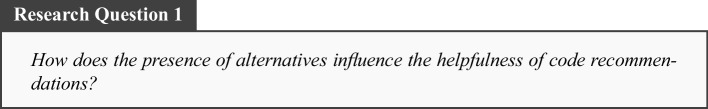


### Solving Data Science Tasks with AI-based Coding Assistants

The rise in LLMs has led to an increasing interest in AI assistance in programming on one side and on the other, equipping developers with prompt engineering skills. These AI assistants for coding are designed to primarily provide functional features without the added complexities of conversation (Barke et al. 2023). While coding assistants can be guided through prompts to generate (alternative) recommendations, we investigate whether leveraging data science step information in prompts can guide an AI assistant in providing helpful recommendations. Therefore, our second research question is: 
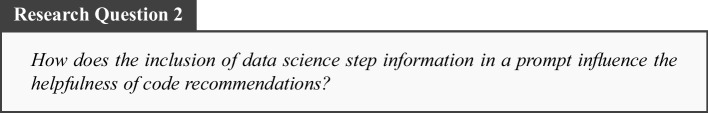


To understand how helpful code recommendations are in two types of common data science tasks — *descriptive* and *predictive* analyses, we investigate the third research question: 
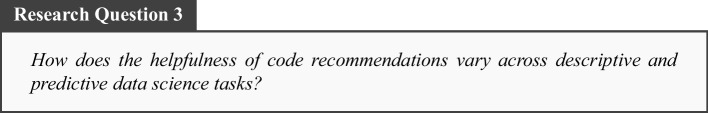


Additionally, we investigate whether users are likely to request a higher number of recommendations at specific stages of a data science workflow. This leads to our fourth research question: 
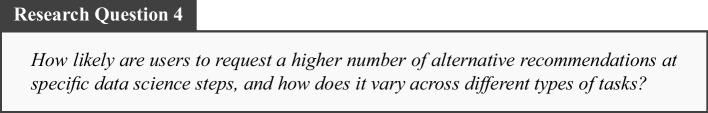


### Interfaces for Coding Assistants in Computational Notebooks

To conduct the study, we designed and implemented a Jupyter plugin that integrates the AI assistant within the Jupyter Notebook.

In the absence of an appropriate interface to interact with the AI assistant, users use code-based API calls to get a response from AI assistants. While there has been interest in exploring the integration of coding assistants within traditional Integrated Development Environments (IDEs) (Ross et al. 2023; Nam et al. 2023), similar endeavours in the context of computational notebooks remain relatively unexplored. In their investigation of the integration of coding assistants within notebooks, McNutt et al. (2023) explored the design space that includes the components (e.g., code) of the notebook environment and how users interact with them through an interview study. They show that the existing interfaces for coding assistance in notebooks are model-specific to an AI system (e.g., GitHub Copilot) and lack cell-based coding assistance (McNutt et al. 2023). Furthermore, existing tools are targeted towards generic coding assistance.

In this study, we develop ***C****ollaborative* ***A****ssistan* ***T*** *for Data* ***Sci****ence* (*CATSci*), a Jupyter plugin that integrates AI assistants in the computational notebook and provides interface elements that are cell-based and model-agnostic. To the best of our knowledge, *CATSci* is the first interface designed specifically to integrate coding assistance in the context of data science. Our design is motivated by data science workflows and provides relevant features in the context of data science tasks. Particularly, the interface has features allowing users to specify the data science step while requesting recommendations and also allowing them to receive alternative recommendations.

## Methodology

To answer the research questions set out in this study, we designed a user experiment where users solve different data science tasks with the help of an AI assistant. The experiment focused on studying three dimensions defined in our research questions: *type of the request* for the recommendation, *presence of data science step information* in the prompt, and the type of the *data science task* and their effect on the helpfulness of a code recommendation measured by two target variables: *acceptance* and *performance* of the recommendation. We explain them below:

### Independent Variables

The study dimensions, controlled in the experimental conditions, are:

 The prompt request may be one of the following two types of requests for recommendation:

: requests *a new recommendation* for the prompt input
: requests *a new recommendation* for the prompt input and *explicitly* requests its *alternatives*

Following the literature (refer to Section 2.3), in this experiment, we define an alternative to be method-based. That is, with respect to the method recommended in the main recommendation, three alternatives that offer other possible methods to choose from are offered to the users.

 Each prompt may contain the  information (  or  ) indicating the step for which a recommendation is requested. We use the categorisation of data science steps in computational notebooks proposed by Ramasamy et al. (2022).

 To understand the recommendation need better, we conduct the study across two types of data science tasks:  and  . A descriptive task summarises, explores, or visualises the data collected to, for example, find patterns. A predictive task uses methods like learning algorithms to make predictions on future data by inferring insights from past data.

### Dependent Variables

In order to evaluate whether a code recommendation is helpful, we measure two dependent variables:

of the recommendation, measured through user’s accept — explicit or implicit — decision for a recommendation by the AI. We measure acceptance by observing the recorded user actions in the log.
of the recommendation, measured by the score assigned to the tasks. The score is determined using an answer key and a scoring mechanism that evaluates the correctness of the final solutions submitted for each task.

We chose the variables to comprehensively capture both subjective and objective aspects of the code recommendations. Acceptance captures the subjective aspect and reflects user’s satisfaction with code recommendations in terms of relevance, usability, and quality. Performance captures the objective aspect, measuring the correctness of the final solutions achieved using the recommended code. The measurement of the two variables can differ: for example, a recommendation might be highly accepted by users for its simplicity but may not lead to the correct solution. By evaluating both metrics, we aim to gain a holistic understanding of how recommendations are perceived and how effective they are in providing a solution to the tasks.

We hypothesise that a helpful code recommendation is generally perceived as both acceptable by the user and also supports the user in accomplishing the task successfully.

Table 1 lists all the independent and dependent variables in the experiment.

**Table 1:** Variables in the experiment

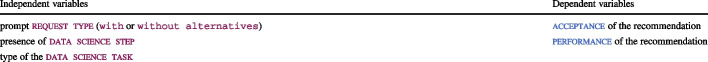	

### Experimental Conditions

The study follows a mixed-method strategy. For prompt  and presence of  , the study is designed as between-subjects leading to four experimental groups. Whereas for the  , we conduct the study within subjects and randomise the order of the type of the task. Refer to Table 2 for the experiment groups. The participants belong to one of the following groups:

**Table:** 

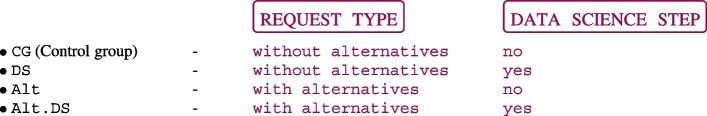

**Table 2:** Experimental Design: 2x2x2 setup of prompt  over presence of  leading to four experimental groups across two types of

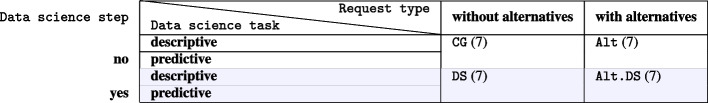	

**Fig. 1:**
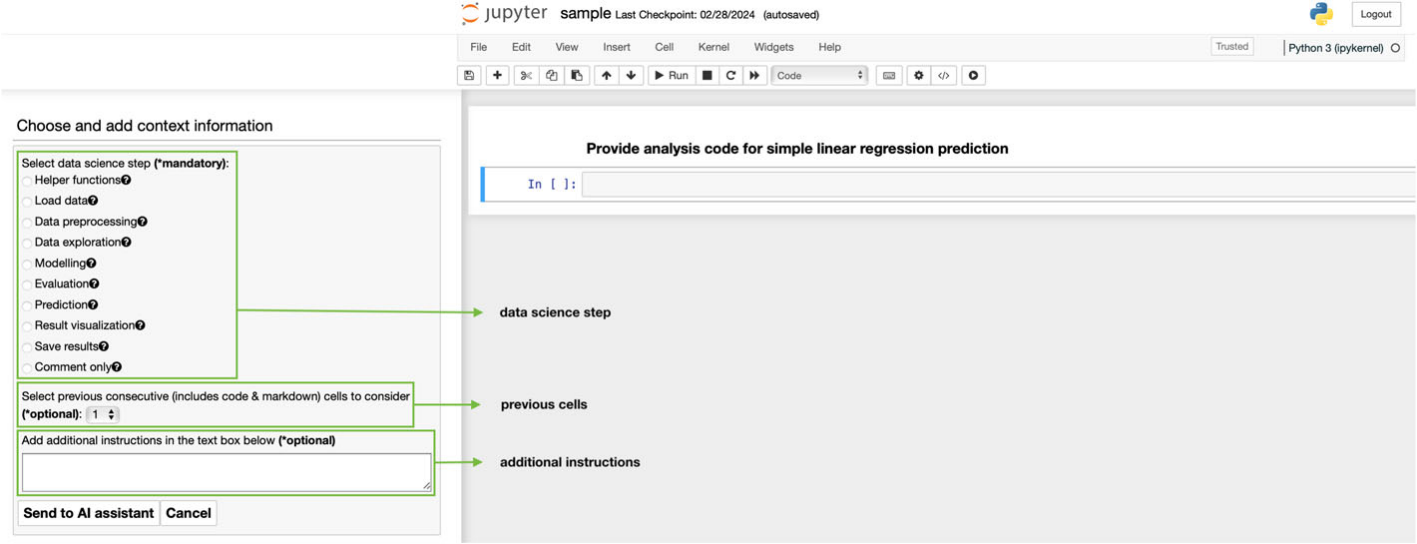
*CATSci* plugin and its for providing prompt input

In the next section, we explain the system we designed and implemented to conduct the study.

## System: CATSci - Interface for Data Science Recommendations

Existing interface extensions to integrate coding assistants for computational notebooks to interact with AI assistants are not tailored towards data science workflows and lack the features necessary for the study. Particularly, we explore features required to control both the input (i.e., providing data science step information in the prompts) and the output (being able to request alternative code recommendations). As a result, we design and implement a cell-based, model-agnostic interface in the form of a Jupyter plugin, ***C****ollaborative* ***A****ssistan* ***T*** *for Data* ***Sci****ence* (*CATSci*).

*CATSci*’s implementation is motivated by Human-AI interaction guidelines (Amershi et al. 2019) and is available as a Jupyter plugin. It is implemented in a non-intrusive way, that is, not interfering with the users’ original notebook and allowing interaction with the AI assistant through a side panel (refer to Fig. 1). Unlike existing tools (Kery et al. 2020; Drosos et al. 2020; hex 2023; cop 2023; tab 2023), *CATSci* supports cell-based interaction as cells are the logical unit of computation in Jupyter. It supports features particularly aimed at data science workflows.

*CATSci*’s interface provides a functional interface to interact with an AI assistant to request the next (step) recommendation for a code cell and allows the users to customise their prompting. By changing the setting in the plugin, it supports the presentation of recommendation responses  or  . CATSci allows the scope of the alternatives (what qualifies as an alternative for a given context and the number of alternatives) to be defined through prompt engineering.

### Prompting through *CATSci*

While there are no established ways to design prompts (Zamfirescu-Pereira et al. 2023), prompts for a coding assistant can generally include information such as code blocks, output data, metadata, and natural language text (McNutt et al. 2023). For data science tasks, we consider the following relevant context information:

1. *data science step:* step in a data science workflow as metadata (based on Ramasamy et al. 2022) in order to indicate the step for which the code recommendation is requested.
2. *previous cells:* previous cells (including code and markdown cells) that are considered to provide context for the next code recommendation (similar to ongoing conversations with the flexibility to select the number of previous cells^1^). By default, the value is set to include all previous cells.
3. *additional instructions:* users can add natural language text to provide more instructions to the assistant.

All of the aforementioned information can be entered through CATSci’s interface. For example, if the user requests a recommendation for a code cell that already contains a code block, the user can choose to include the existing code block in the context for prompting. Figure 1 shows the user interface that allows the user to enter all the information for the prompt.

### Receiving Recommendations through *CATSci*

**Fig. 2:**
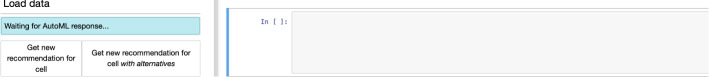
*CATSci* interface for recommendation type. Users in  group receive only the ‘Get new recommendation for code cell’ button, whereas the group  receive only the ‘Get new recommendation for code cell *with alternatives*’

*CATSci* supports receiving recommendations  and  . When it is set up  , then *CATSci* requests the AI assistant to return one response. When it is set up  , then *CATSci* requests the AI assistant to return a response along with three alternatives. For easier prompting in the case of data science tasks, the user interface supports the above types through the click of a button (refer to Fig. 2).

**Fig. 3:**
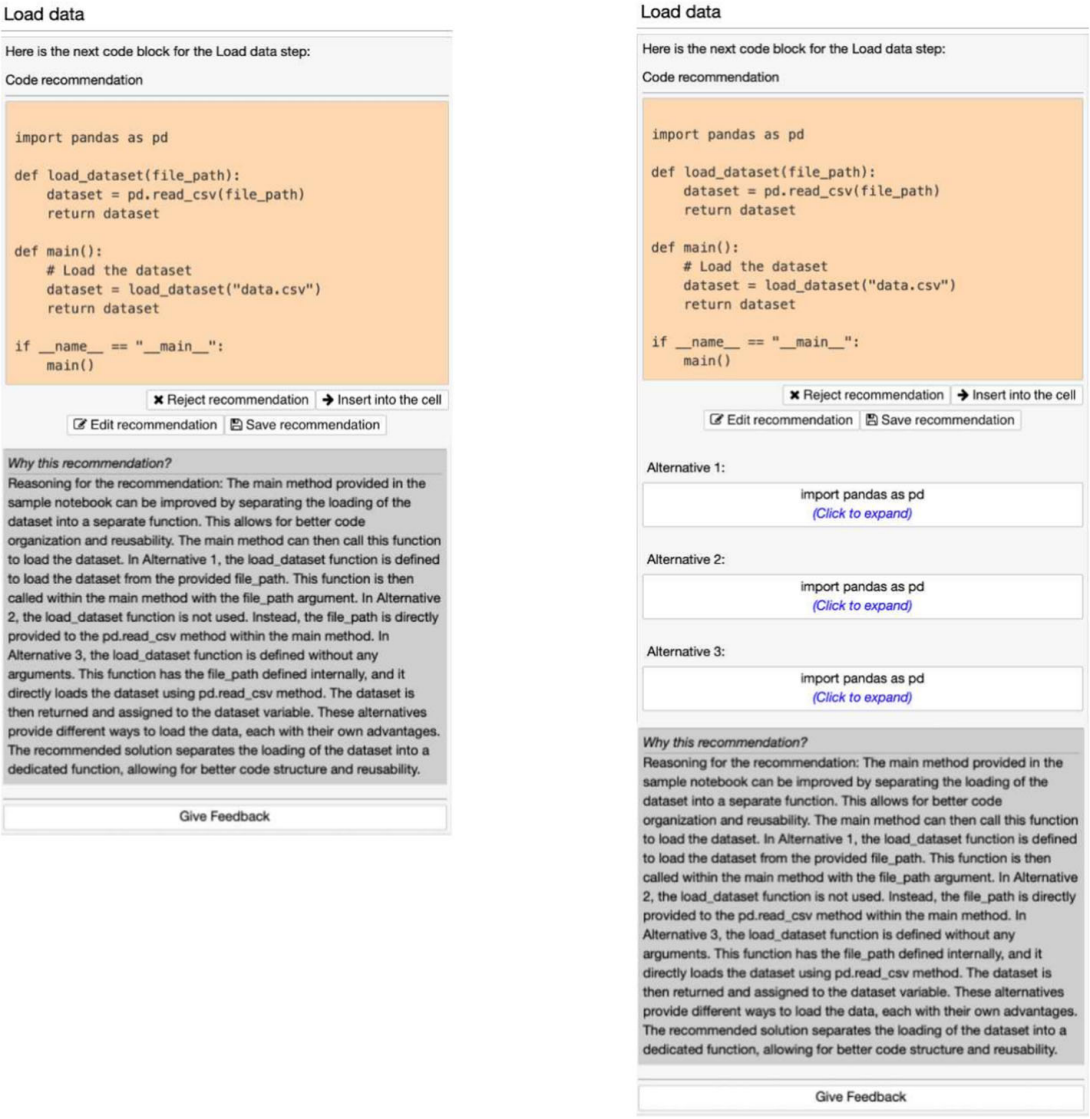
An example recommendation output for  and  request is shown on the left and right respectively. In the case of alternatives, the user is provided with several alternatives to be explored further

For presenting code recommendations to the user, *CATSci* uses the side-panel space that mimics a code cell to show the recommended code. *CATSci* processes the response from the model’s API and presents it based on the prompt type. Through the pre-defined prompt template, we guide GPT’s response to follow a consistent format in order to process the response. The example output for the two prompt types — with and without alternatives — is shown in Fig. 3. Currently, *CATSci* supports prompting and processing the response of the ChatGPT API. By adding further processing support, *CATSci* can be easily extended to support other AI models.

The user can interact with the code recommendation using the following actions: *accept* the recommendation, *reject* the recommendation, *edit* the recommendation, and *save* the recommendation for later use. If the user is happy with the recommendation, they can insert it into the cell with a click of a button or reject it using the corresponding buttons. By showing the recommendation in the sidebar, ***C****ollaborative* ***A****ssistan* ***T*** *for Data* ***Sci****ence* allows the user to inspect or edit the code before integrating the recommendation into their workflow. The user can also name and save the recommendations for later reference and use. Additionally, the user can provide feedback on the recommendation through likes, dislikes, or a text comment.

### An Example user Journey using CATSci Interface

We present a simple scenario to exemplify the user journey using the *CATSci* interface in computational notebooks for code recommendations  and  . Let’s call the user F.

**Fig. 4:**
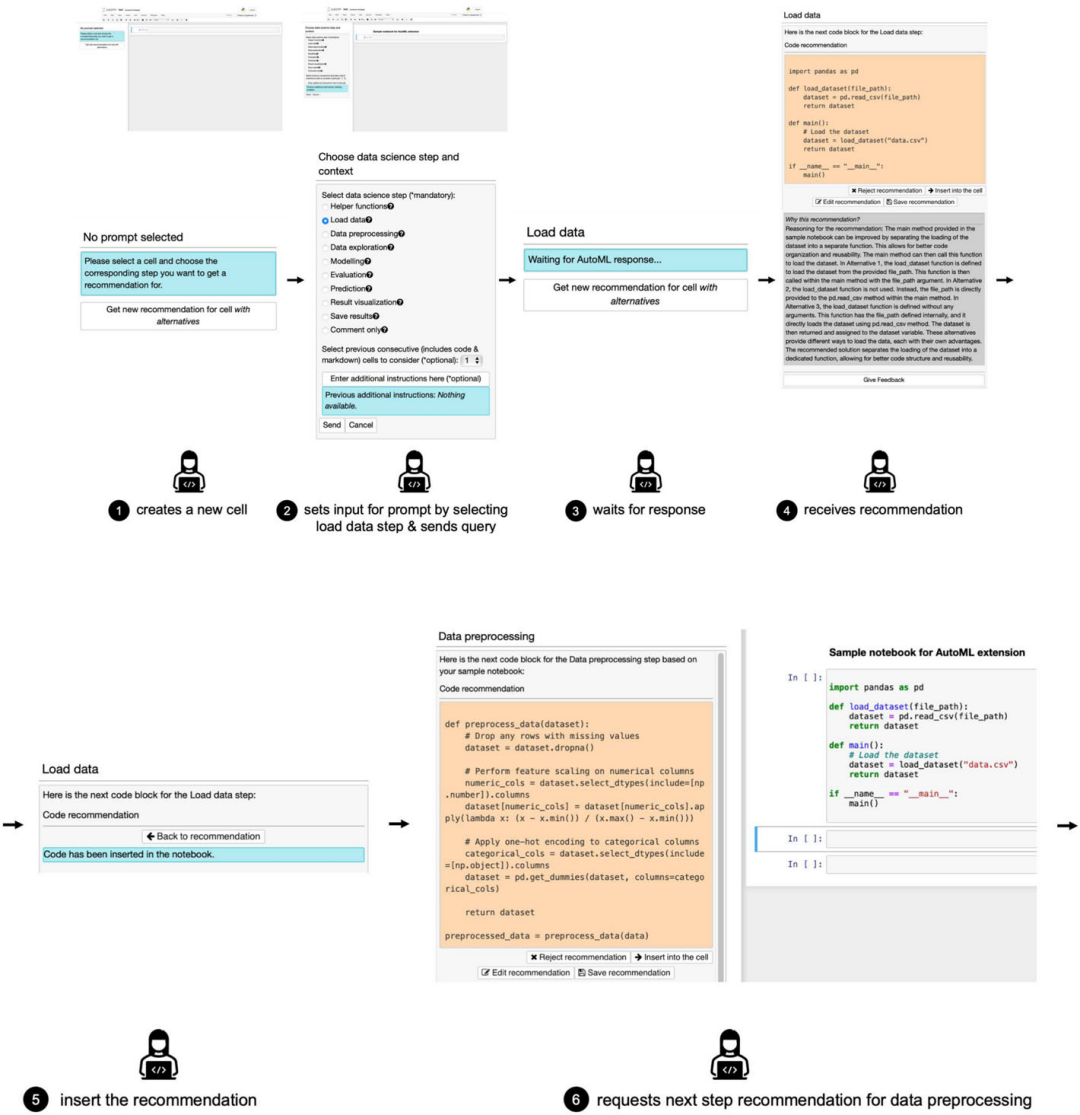
User journey with *CATSci* interface for recommendations

❶ User F would like to implement a new data science task and therefore opens a Jupyter notebook, which has *CATSci* configured to work without alternatives. Now, F would like to get some help writing the code in order to load data into the notebook. ❷ F decides to request a recommendation through *CATSci* by setting the data science step as Load data in the input for prompt through CATSci’s interface and sends the prompt query. ❸ *CATSci* creates and sends the pre-defined prompt based on the input parameters through ChatGPT API. ❹ The response received is processed and presented by *CATSci* for F. F can accept, reject, and edit the recommendation before incorporating it in the notebook cell and save the recommendation for future reference. F evaluates the recommendation and decides to accept the recommendation without further changes. ❺ F inserts it into the cell through a simple button click and integrates the recommendation in the notebook. Now, F would like to preprocess the data. F selects the Data preprocessing step in the input parameters and sends the prompt query. ❻ *CATSci* now presents the response received for F. F can now continue to interact with the AI assistant easily through *CATSci* in order to complete the task. The user journey is shown in Fig. 4.

**Fig. 5:**
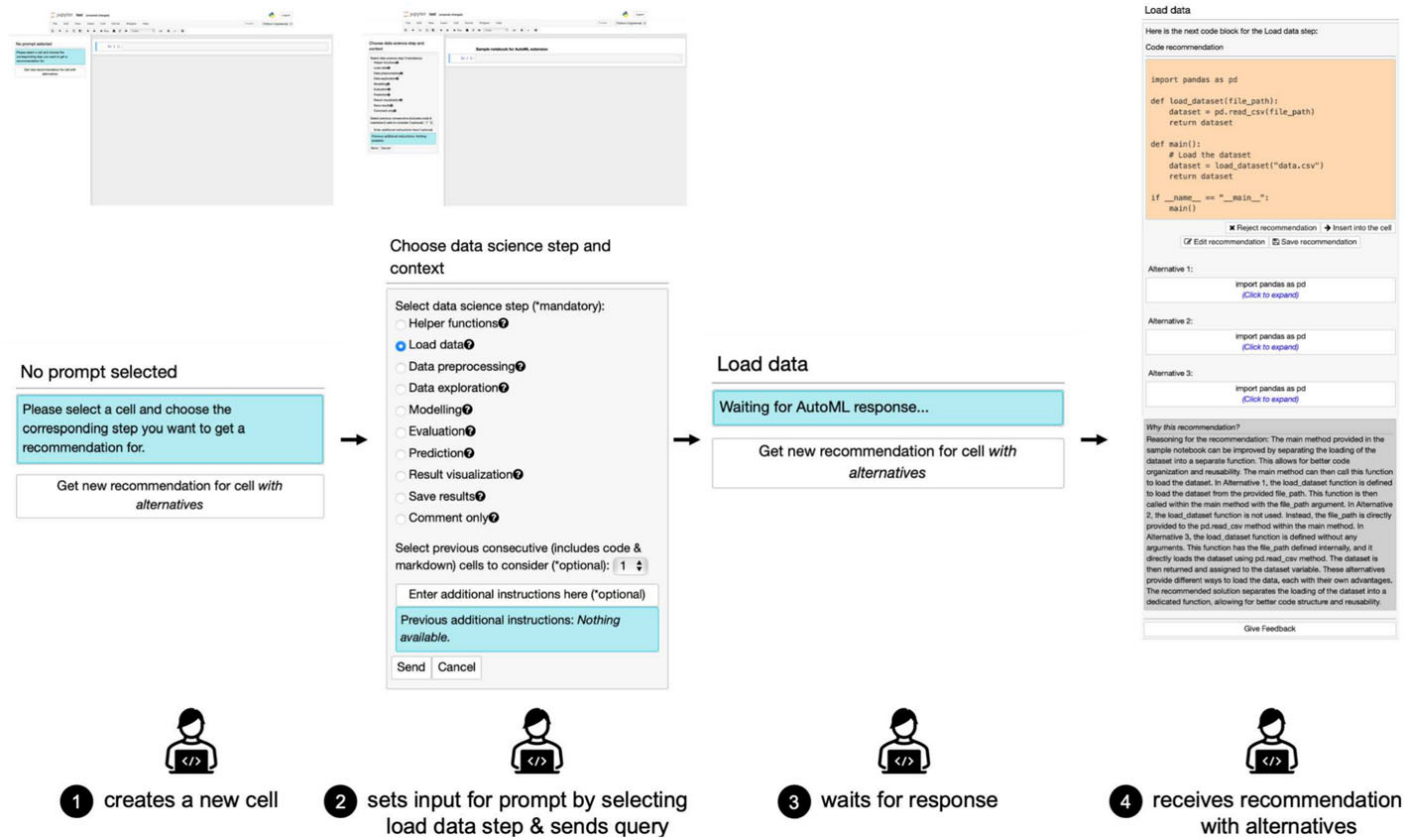
User journey with *CATSci* interface for recommendations

By changing the setting for ***C****ollaborative* ***A****ssistan* ***T*** *for Data* ***Sci****ence* to work  , F can send a request to receive recommendations that also contain several alternatives to a given recommendation. This scenario is shown in Fig. 5.

## User Experiment

### Experiment Design

We conducted a controlled experiment with data scientists, in which they completed different types of data science tasks (i.e., descriptive and predictive tasks). Data scientists solved the tasks in Jupyter and interacted with the AI assistant through CATSci to get recommendations for the next step they would like to take in the workflow. After this, they answered a set of questions about their experience. The entire study was guided by a survey. We provide the details of this empirical study in this section.

### AI Assistant

We selected OpenAI’s GPT-4 model as it is one of the most used and popular (Zamfirescu-Pereira et al. 2023; McNutt et al. 2023) baseline models in handling a broader range of tasks. We selected the GPT-4 model for the main experiment as it is one of the latest and has been expected to be superior to GPT-3.5.

#### Alternatives

Through *CATSci*, we guide the GPT model to generate alternatives to the main recommendation through prompt engineering. In order to avoid overwhelming the user, only the top three alternatives are requested. If a new recommendation is requested, the treatment group is then provided with a set of alternatives relative to the new recommendation (refer to Fig. 3). When data science step information is present in the prompt, the model is prompted to provide alternatives that offer other possible methods for that *particular step*.

#### Pre-defined Prompts

**Fig. 6:**
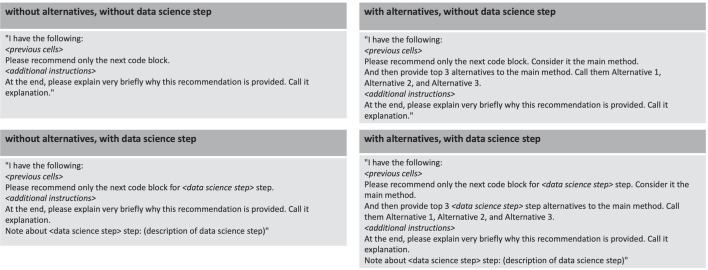
Pre-defined prompts for the different experimental conditions of  and presence of

As the task is always to generate the next code block, we use different pre-defined prompt templates tailored for different experimental conditions. These templates were carefully developed based on experimentation in order to optimise for the *CATSci* interface and user experience. By incorporating these pre-defined prompts into *CATSci*, users can efficiently request recommendations without the need to repeatedly write prompt text. Additionally, *CATSci* offers flexibility by allowing users to customise pre-defined prompt templates based on their preferences through the context information element (refer to Fig. 1). Users can incorporate relevant context information into these templates before they initiate requests (refer to Fig. 2). For example, when a data science step is selected by the user through *CATSci* interface, a description of that data science step (as provided by Ramasamy et al. 2022) is added to the prompt. Refer to Fig. 6 for the different pre-defined prompt templates used in the experiment.

Below, we provide a concrete example of a user-customised prompt for the experiment condition without alternatives and without data science step:

I have the following:

import numpy as np

Please recommend only the next code block.

I want to load pandas.

At the end, please explain very brefly why this recommendation is provided. Call it explanation.

### Interaction Log

In addition to the dependent variables (refer to Table 1), for each user-*CATSci* interaction, we record the following information by logging the interaction (based on the variables defined in Section 3.2) to support our analyses:

**request:** the prompt text along with its request type.
**accept/reject:** if the code recommendation is accepted or rejected explicitly.
**auto-reject:** if the code recommendation is neither accepted nor rejected.
**edits:** if the code recommendation is edited before integrating it into the notebook workflow. We account for this action as an auto-accept due to a user’s willingness to interact with the code.

Furthermore, we record the solution to each data science task and record the status of the notebooks by taking a snapshot of the notebook file every minute for later verification if required.

### Pilot Study

We ran several major pilot studies to test and improve the instructions and the experiment setup before running the main experiment. First, we ran a preliminary study with researchers from our lab. Then, for our next pilot, we recruited external data scientists from Prolific. We selected Prolific users who were 18-65 years old and knowledgeable in computer programming and Python. When users signed up for the study, we provided them with a survey asking them to self-report their prior experience with data science tasks and Jupyter Notebooks. As the experiment involves working with Jupyter notebooks, we selected those participants who had reported to have experience working with them. We followed Prolific policies for data collection and consent and compensated the participants for their time ($20\$$). Our insights from pilot studies led to several improvements in the instructions for clarity, setup (e.g., providing separate video links since embedding them in the survey can lead to a slow response time), and survey (additional questions to get user feedback). The Prolific setup was inadequate, probably due to the complex nature of the experiment. Therefore, we conducted an additional pilot study using Upwork platform. Upwork allowed us better control over the experimental procedure (e.g., conducting video calls) and helped ensure a more rigorous selection of data scientist participants.

For the main experiment, we used Upwork platform as it provided more suitable options. The remainder of this section details the methodology followed for the main experiment.

### Participants

For the main experiment, we recruited data scientists from Upwork as participants. We used screen recording for observation in order to control the experiment. We recruited those who had self-reported experience of at least one or more years of data science. Once selected, we randomly assigned them to one of the experimental conditions and scheduled a time slot for them to take part in the study. We started with five participants per group in the first round and added two per group in the next round. As the results did not change, the final count of participants was seven data scientists per group. We report the self-reported experience of the participants in data science tasks in Table 3. Participants gave informed consent for anonymised usage of their data for research purposes. At the end of the study, we compensated them for their time and solutions.

During the main experiment, participants were asked questions about their experience with AI assistants. Almost all of the participants had reported previous experience with AI assistants. An overwhelming majority of them reported using ChatGPT for data science and programming in general.

**Table 3:** Number of participants belong to each experiment group based on their self-reported experience in data science tasks

Group	1-5 years	5-10 years
CG	6	1
DS	6	1
Alt	5	2
Alt.Ds	6	1

#### Power analysis

According to G*Power software, for an experiment with four groups across two factors, 3 covariates, an error probability of 0.05, a large effect size of 0.4 (Cohen 2013), and a total sample size of 28, a power of 0.5 is achieved.

### Environment and the Task

#### Environment

We hosted the infrastructure for the experiment on a server at our institution. We provided Jupyter Notebook with *CATSci* plugin to interact with the GPT model enabled for finishing the two data science tasks. Each user was allotted a workspace where they also had access to the data files for the tasks. The survey is conducted using a Qualtrics survey (refer to Supplementary Information for the complete survey).

#### Task

We designed the tasks, descriptive and predictive, based on publicly available data sources (refer to Supplementary Information for complete details). We designed the data science tasks with a focus on simplicity and clarity to ensure that participants could concentrate on the core objectives. These tasks were intended to be straightforward examples, effectively demonstrating how AI-generated code recommendations perform in solving data science challenges.

For the descriptive task, participants were given a dataset^2^ taken from the U.S Centres for Disease Control and Prevention (CDC), containing survey data on mental health in the United States. We preprocessed the dataset to reduce the size by removing irrelevant data for the given task, resulting in a total size of 4131 rows and 14 columns. Participants were tasked with identifying the pair of highest correlating states between the given pair of states in the United States and mental health indicators. For the predictive task, participants engaged in classifying features of a financial dataset. They were provided with credit card fraud detection dataset^3^ from Kaggle, containing anonymised information about credit card transactions. They were asked to predict the labels of the test data based on the patterns in training data. The training data set contains 22698 rows and 31 columns while the test data set contains 5675 rows and 30 columns. Before the experiment, we evaluated the performance of the GPT model on both tasks in order to confirm that the API provides a valid set of responses. The dataset and task files provided to the participants are available for open access at https://doi.org/10.5281/zenodo.13639707.

The Supplementary Information contains the instructions including complete task description.

### Experiment Setup and Procedure

The experiment was set up as a guided Qualtrics survey and follows the structure below. The complete protocol of the experiment and the instructions (for the group Alt.DS that includes all the conditions) are provided in the Supplementary Information. Before conducting the study, we received ethics approval from our institutional ethics board for both the pilot and main experiment.

During the scheduled slot, the participants took part in the experiment moderated through screen-sharing (no audio and video of participant) on a Zoom call in Upwork. The screen-sharing was used for observational purposes to resolve any technical issues and was not recorded.

First, users were informed of the nature of the experiment and data collection and were asked for their explicit consent. Then, they followed the instructions provided in the survey in three parts.

#### Pre-task Questionnaire

In the first part, they answered a set of questions about their experience with AI assistants for programming.

#### Introduction to the *CATSci* Interface

In the second part, the participants received information about the *CATSci* environment based on their experimental condition. Then, they watched a video that explained the Jupyter Notebook environment along with functionalities available through the interface in detail. At the end of it, users received the link to the Jupyter Notebook and had five minutes to play around with the interface before the main tasks started.

#### Solving the Data Science Tasks

The users then received the tasks to solve using the Jupyter Notebook environment provided. We randomised the order of the tasks provided in order to control for possible learning effects. The participants followed their development process within the Jupyter Notebook without further restrictions. That is, activities such as shifting away from the browser within the scope of the task and searching online materials relevant to the task were not explicitly disallowed. In the instructions, we asked the participants to use the AI assistant as much as possible to solve the tasks instead of writing their own code from scratch. At the end of each task, users submitted their solutions and answered a set of questions related to their solution (confidence in the solution, if the solution contains interpretable methods, and if the solution is understandable for someone without their help) by recording them in the survey.

#### Post-task Questionnaire

In the last part, participants answered a set of questions about their experience (refer to Supplementary Information). The questions were aimed towards understanding i) users’ perspectives on their analytical solutions to the tasks, ii) recommendations provided by the AI assistant and their interaction experience with the AI assistant, and iii) usability of *CATSci*. First, we collect feedback related to the recommendations that form the major focus of our study. Next, the questions focused on the features of the interface and the usability (adapted from System Usability Score Brooke 1996).

### Hypotheses

In order to answer the research questions, we tested several hypotheses that we list in Table 4.

**Table 4:** Hypotheses associated with the research questions

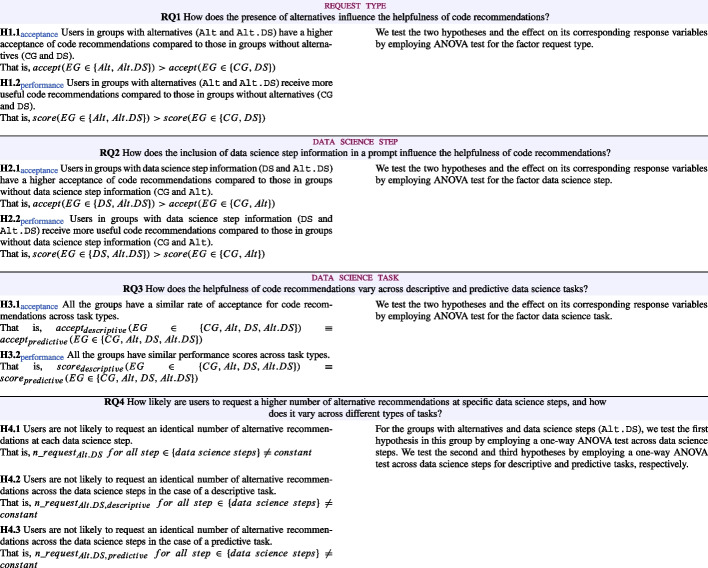	

### Data Collection and Analysis

We collect the interaction log data (refer to Section 5.3) and the survey results (refer to Section 5.7) in our experiment. For the inferential analysis of interaction log data, we employ an analysis of variance (ANOVA) test. For the analysis of the survey results, we employ both quantitative and qualitative methods. In the case of qualitative analysis of results from open-text questions, we employ an in-vivo coding strategy.

## Results

In this section, we report the quantitative analysis based on the hypotheses we set out to study. We publish the data in an anonymised format along with the analyses here: https://doi.org/10.5281/zenodo.10943330. The  — average probability of acceptance of recommendation — and  — average scores achieved on tasks — are reported in Table 5.

**Table 5:** Average probability of acceptance of recommendations (  ) in % and average scores achieved on tasks (  ) across groups presented in the format (  ,  )

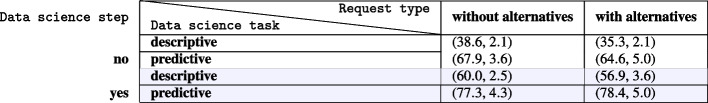	

We conducted a three-way ANOVA test for the main effects across the three dimensions for both cases of dependent variables, as we found no significant interaction effects. The result shows that there are *statistically significant* differences among the groups for both  ($F = 6.74, p = 0.001$) and  ($F = 6.46, p = 0.001$). The model result shows that:

as target variable: On the target variable  ,  and  have *statistically significant* effects with values $F = 6.36, p = 0.015$, $\eta _{p}^{2} = 0.09$ and $F = 13.74, p = 0.001$, $\eta _{p}^{2} = 0.19$, respectively. Groups that have  have a *statistically significant* higher probability ($+16.57\%$) of  of a code recommendation in comparison to groups has no data science steps. Predictive  has a *statistically significant* higher probability ($+24.36\%$) of  of a code recommendation in comparison to descriptive tasks.
as target variable: On target variable  ,  has a *statistically significant* effect with value $F=14.97$, $p=0.000$, $\eta _{p}^{2}=0.21$. Predictive  has a *statistically significant* higher average score ($+1.88$), that is,  in comparison to descriptive tasks.

A post-hoc Tukey’s test shows that there is a *statistically significant (adjusted p = 0.0493)* difference between Alt group descriptive task and Alt.DS predictive task with the latter having $+43.14\%$  .

In order to answer the hypotheses related to the research questions, we conducted an ANOVA test across the factors. The results along with whether the hypotheses are supported are summarised in Table 6.

**Table 6:** Hypotheses results

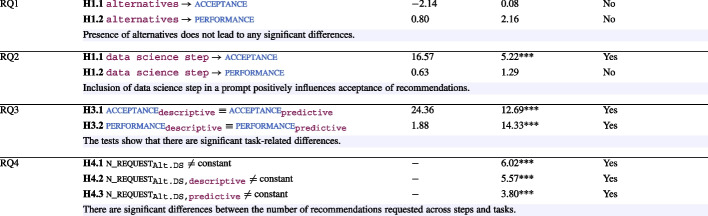	

*** indicates $$p < 0.05$$

Based on the results from Table 6, we summarise the findings of RQ1-3 below: 
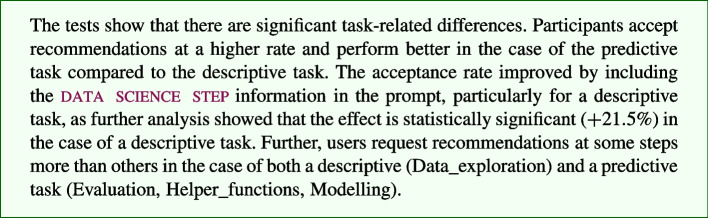


For *RQ*4, we investigated further to get a fine-grained insight into Alt.DS, which performed the best in terms of both the dependent variables among all the groups.

As we found statistically significant evidence in our one-way ANOVA analysis that users do not request an identical number of (alternative) recommendations across data science steps, a post-hoc analysis was conducted. The results reveal that Evaluation ($-4.29$), Helper_functions ($-4.89$), Prediction ($-5.0$), Result_visualization ($-5.43$), and Save_results ($-5.29$) all have lower number of requests than Data_exploration.

**Fig. 7:**
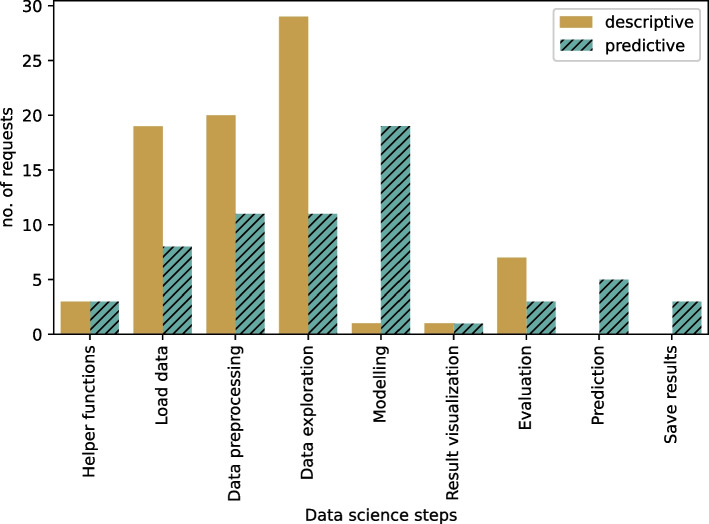
Number of requests across steps and tasks

Furthermore, for both the descriptive and the predictive tasks, we found statistically significant evidence that users do not request an identical number of alternative recommendations across data science steps. Figure 7 shows the number of requests across the data science steps for the two data science tasks. Unsurprisingly, the descriptive task has the highest number of requests for Data_exploration, whereas the predictive task has the highest number of requests for Modelling. This result is confirmed to be statistically significant in the post-hoc analysis.

The post-hoc analysis revealed that Evaluation ($-3.14$), Helper_functions ($-3.71$), Modelling ($-4.0$), Prediction ($-4.14$), Result_visualization ($-4.0$), and Save_results ($-4.14$) all have lower number of requests than Data_exploration in the descriptive task. For the predictive task, a post-hoc analysis revealed that Evaluation ($+2.29$) and Helper_functions ($+2.29$) have more requests than Modelling. Prediction ($-2.0$), Result_visualization ($-2.57$), and Save_results ($-2.29$) all have a lower number of requests than Modelling.

### Between Group Analysis

Not considering the task levels, we tested whether the users in the group with both alternatives and data science step information (Alt.DS) have a higher acceptance of code recommendations (  ) and receive more useful code recommendations, that is, it leads to better score (  , refer to Section 3.2) compared to those in groups without either or both. That is,

$$\begin{aligned} \begin{aligned}&P(accept | Alt.DS)> P(accept | any \;from\;\{CG, Alt, DS\}) \\&\, P(score | Alt.DS) > P(score | any \;from\;\{CG, Alt, DS\}) \end{aligned} \end{aligned}$$

We find  had significant effect ($F=5.13$, $p=0.028$) on  ($+16.57\%$). A post-hoc analysis does not show any difference among groups.

### Discussion

Our result shows that including the information about  while prompting can generate a recommendation that has a significantly higher probability of  . In practice, this can have several advantages. First, guiding the model to generate more relevant recommendations by taking into account the additional context of the data science step. Second, it reduces user frustration by minimising the need for multiple prompt requests to obtain an acceptable recommendation. Third, it has the potential to accelerate the development process, leading to increased productivity benefits.

For  with alternatives, while our observation shows that participants were intrigued by their presence, there is no statistical evidence on their  . We find that, in groups with alternatives, a total of $91\%$ of the main and $9\%$ of alternative recommendations were accepted. For Alt, $88.0\%$ main recommendations and $12.0\%$ alternatives were accepted, whereas for Alt.DS, $94.0\%$ main recommendations and $6.0\%$ alternatives were accepted. This shows an increased acceptance of main recommendations when there is an inclusion of a data science step. On  , while not statistically significant, Alt.DS achieves the best score in the descriptive task, and the groups with alternatives (Alt and Alt.DS) achieve the best score in the predictive task, showing the potential of alternatives.

**Table 7:** Number of descriptive and predictive requests made across experimental groups

Group	Descriptive	Predictive	Total
CG	124	87	211
Alt	131	71	202
DS	76	72	148
Alt.DS	80	64	144

When it comes to different types of  s, our analysis shows that there are significant differences in terms of both  and  . Code recommendations for predictive tasks are more accepted than the code recommendations for descriptive tasks (refer to Table 5). Also, further analysis of the interaction log shows that more requests were made for the descriptive task than the predictive task across all the groups. The number of requests among the different types of tasks across the groups is provided in Table 7. The number of unique requests (in descending order) made by each group are 211 for CG, 202 for Alt, 148 for DS, and 144 for Alt.DS. The highest number of unique requests were made by the control group (CG), and the lowest number of requests were made by the Alt.DS group. This shows that the control group (CG) made more requests by interacting with the AI model to obtain acceptable recommendations for successfully solving the tasks, potentially reflecting a higher level of effort or difficulty in the process. Also, results in Table 5 show that recommendations are significantly more effective in assisting users to accurately address predictive tasks compared to descriptive tasks. This shows the state-of-the-art model’s limitations in generating acceptable and effective recommendations for descriptive tasks. However, by including the data science step information in the prompt, the number of requests to obtain acceptable recommendations can be reduced to a large extent (refer to Table 7).

## Survey Analysis

In this section, we report the results of the post-task questionnaire survey answered by the participants (7 per experimental group).

The post-task questionnaire consisted of various parts and is presented in the sub-sections below in the order of appearance in the survey. We discuss both the quantitative and qualitative results for each of these parts and elaborate on the relevant open-text comments provided by the participants.

### Solving Data Science Tasks with AI Assistant

At the end of completing each task presented, participants were asked how confident they were with regard to their solution and whether their solutions contained interpretable methods and were understandable to a third party without their help.

#### Confidence

**Fig. 8:**
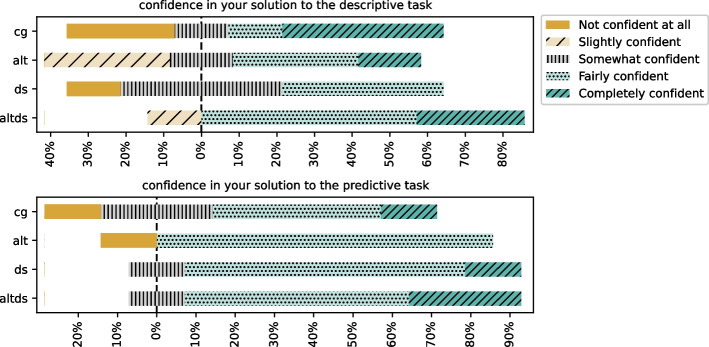
Confidence reported by the participants

Users reported their confidence on a Likert scale for each of the tasks they solved. Self-reported scores (refer to Fig. 8) show that participants are generally more confident about their solution to the predictive task rather than the descriptive task. In the descriptive task, having both alternative and data science steps helps improve participant confidence.

#### Interpretability & Understandability

$85\%$ or higher number of participants rated their solutions to both descriptive and predictive tasks as containing interpretable methods and understandable for others.

### Data Science Code Recommendations by AI Assistant

#### Requesting the Recommendations

**Fig. 9:**
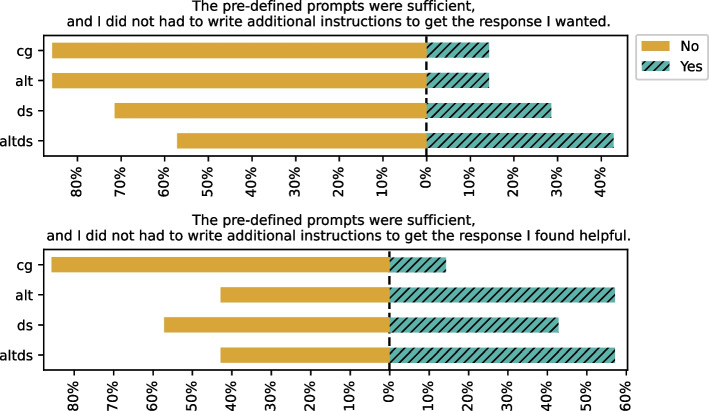
Prompting preference

In this section, we discuss participants’ views on prompting and their preferences regarding various context information that can be added to the prompts. In Table 8, we report the results regarding participants’ preferences regarding context information. The results show that the most popular ($\approx 82\%$ of participants) context information across all groups was adding additional instructions to guide the recommendations. Further, Fig. 9 shows that participants felt more need for additional instructions in the descriptive task than in the predictive task. Notably, for CG group, the need remained constant for both tasks. Other context information, such as adding previous cells and data science step (when available), were equally popular ($\approx 64\%$) on average across groups.

**Table 8:** Percentage of participants’ preference to send each of the context information in a request

Group	previous cells	additional instructions	data science step
CG	42.86	85.71	-
DS	100.00	100.00	85.71
Alt	71.43	71.43	-
Alt.Ds	42.86	71.43	42.86

**Fig. 10:**
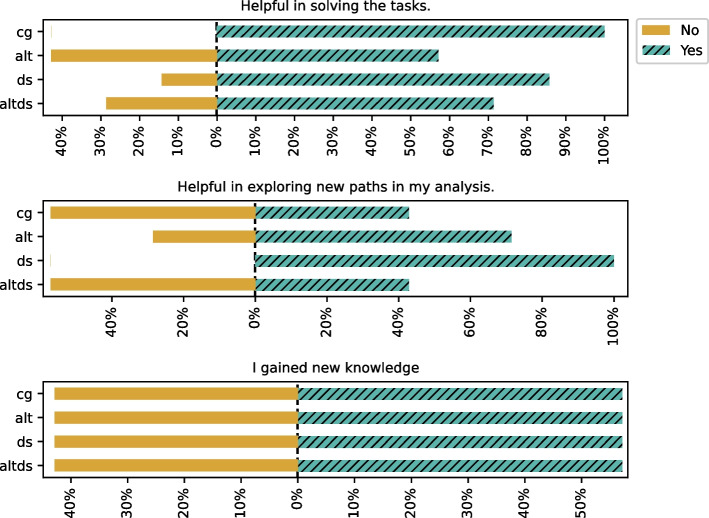
Evaluating the recommendations on their utility

**Fig. 11:**
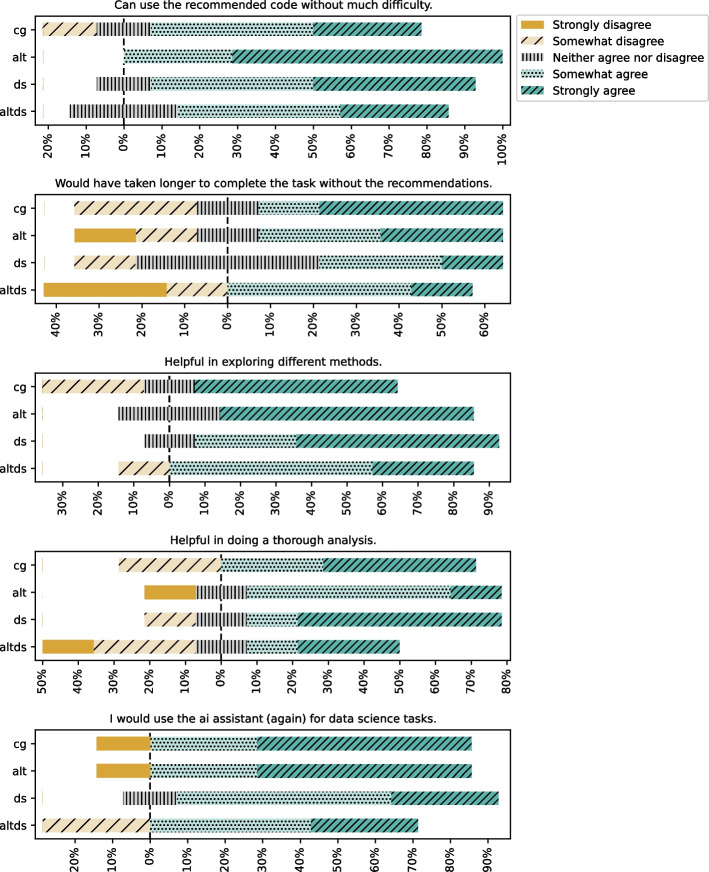
Using the recommendations

#### Evaluating the Recommendations

The recommendations met the expectations of ($\approx 57\%$) of the participants in terms of both content and quality. The CG group and Alt group reported $\approx 100\%$ in terms of content and $\approx 57\%$ in terms of quality, whereas groups with data science step reported $\approx 86\%$ in terms of content and $\approx 71\%$ in terms of quality. When specifically asked about alternative recommendations, $\approx 71\%$ of the participants with alternatives rated the recommendations in terms of content and $\approx 57\%$ in terms of quality. In the presence of the data science steps, this improves to $\approx 86\%$ in terms of content and $\approx 71\%$ in terms of quality. This result shows that the presence of data science steps improves the quality perception of a recommendation, and the presence of alternatives improves content perception.

When asked about the utility of recommendations (refer to results in Fig. 10), the majority of the participants rated recommendations as generally helpful in solving the tasks. Notably, participants in DS group rated recommendations more helpful in exploring new paths.

The above set of results shows that participant perception of recommendation is generally in line with the performance exhibited by the groups.

#### Using the Recommendations

We report the results of participants’ views on using the recommendations presented to them in Fig. 11. The questions focused on understanding participants’ views on using the recommendations presented to them and whether they will use the assistant again for data science tasks. Participants generally reported being able to use the recommendations without much difficulty. They also reported them to be helpful in exploring different methods and doing a thorough analysis and generally said they would use the AI assistant for data science tasks. A notable exception was that approximately one-third of participants disagreed that they would have taken longer to complete the task without the AI recommendations.

#### Recommendations for Descriptive vs Predictive Task

**Fig. 12:**
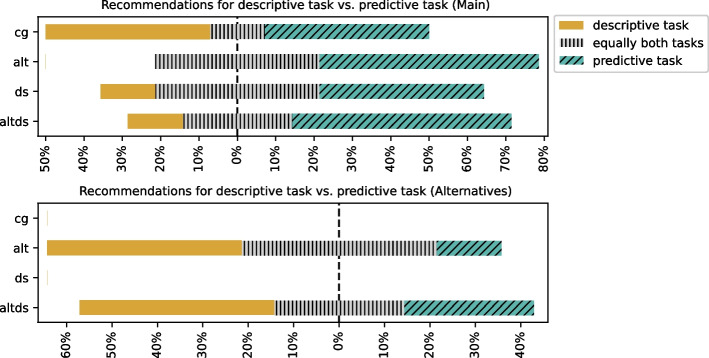
Recommendations for descriptive vs predictive task

The alternative recommendations, when available, are perceived to be more helpful for descriptive tasks, whereas the main recommendation is helpful for predictive tasks (refer to Fig. 12). In the CG group, the recommendations are perceived to be equally helpful for both descriptive and predictive tasks.

#### Reliance on AI Assistance Across Data Science Workflow

In groups that received data science step, participants rated their reliance on AI would be the highest for Evaluation and Prediction steps, scoring above 72 on a scale of 100. Among the top five steps for reliance on AI is also Modelling step. The rest of the top five steps included Load_data and Results_visualization for DS group whereas for Alt.DS group, Data_preprocessing and Data_exploration steps completed the lot. From the interaction log, we find that the highest number of requests were made for Load_data, Modelling, Data_preprocessing, Data_exploration, and Helper_Functions.

##### Discussion

Participants, in general, felt that the recommendations were good. One participant wrote *“It was great that by only knowing the concepts I was able to complete the two tasks with the help of the recommendations.[sic]”* Particularly, they felt it was easier to get the right recommendations for predictive tasks. One participant wrote *“the major positive is the time saved with doing predictive tasks I think that is fantastic and it is a fantastic tool for that.[sic]”* We find this is relevant regarding the comments regarding prompt engineering as well since participants indicated a need for writing detailed prompts, particularly in descriptive tasks. This indicates the difficulties in translating descriptive tasks (possibly providing multi-lingual support) to effective prompts and is a major area to improve in order to provide successful assistance in data science tasks.

Another area of improvement is providing recommendations that follow coding styles that are usually found in notebooks. One participant wrote *“having print statements for dataframes in functions isn’t ideal, I expect less of that. The assistant should do better with function naming and not in all cases should the recommendation be a function.[sic]”* This may be due to the majority of the training data being traditional software engineering code where functions are more common.

Three participants also noted the issues in the code recommended. One wrote *“when I asked to calculate correlations and the code that AI provided was nan. I knew from experience that this is an indexing issue and so I asked for a correcting code in a cell before from AI and then it worked.[sic]”* Two participants said the assistant may help novices or experts (as it requires expertise to spot errors that otherwise might be hard to find without practice). One participant wrote *“I think this is an awesome tool, especially for experts as it does most of the things for us. For someone who is new to programming, it might be difficult to spot some of the logical errors that I encountered that I mentioned before.[sic]”*. This is also reflected in the comments that ask for ‘Comprehensive assistance’ that includes debugging abilities, managing packages, interpretation etc. For experts, the plugin takes off a lot of time from writing redundant, simple code blocks (also indicated by the comments on ‘Ability to complete tasks with less focus on syntax’).

Two participants mentioned that recommendations should not suggest libraries that were not already installed in the system or automatically install them before the subsequent code. This would mean sandboxing the execution before recommending them to the user. Participants also mentioned they want more ‘useful’ alternative recommendations and expect recommendations to provide helpful links or information on models/modules.

### Interface to Interact with AI Assistant

#### Interface Features

**Fig. 13:**
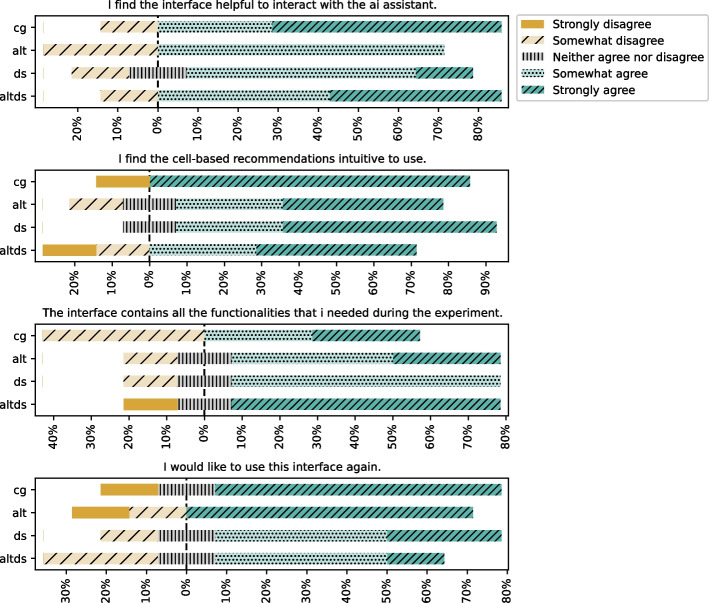
Feedback on the interface to interact with the AI assistant in Computational notebooks

The survey responses show that the majority of the participants find that the interface is helpful and easy to use. They generally agree that cell-based recommendations are intuitive to use and contain necessary functionalities (refer to Fig. 13). However, they also had some concerns, which we elaborate on in the discussion.

#### System Usability Score (SUS)

**Fig. 14:**
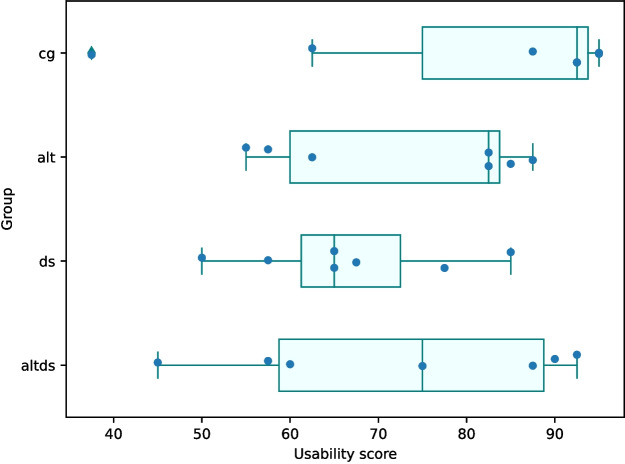
System Usability Score (SUS) for the CATSci interface by groups (scores of each 7 participants per group are identified by markers)

The scores on the system usability test (adapted from Brooke 1996) for the groups were: CG - 80.36, DS - 66.79, Alt - 73.21, Alt.DS - 72.5, and averages to *Good* usability on the adjective rating. Please refer to Fig. 14 to find the scores of each participant in the four experimental groups (7 participants per group) and their distribution. Our results show that having additional elements in the interface marginally diminishes the overall usability scores, particularly in the case of data science steps. This is in contrast to the performance achieved by the corresponding groups (refer to Section 6).

##### Discussion

The quantitative results show that all the treatment groups performed better on average than the control group. Furthermore, the data science steps produced a statistically significant improvement in leading to acceptable code recommendations (refer to Section 6). Our analysis of the interaction log also showed that the presence of data science steps led to $\approx 29\%$ fewer requests and higher acceptance of main recommendations in groups with alternatives — $88\%$ in Alt and $94\%$ in Alt.DS. Additionally, participant perceptions of recommendations were generally favourable in treatment groups, particularly with the data science steps (refer to Section 7.2). Based on the participants’ feedback, participants with the data science steps reported benefits and also difficulties. We discuss the participant feedback below to provide more insights.

In general, participants felt the *CATSci* interface was simple and easy to use. Many participants appreciated the context elements in general, and some also mentioned that they felt adding previous cells gave them better recommendations. One participant wrote *“Providing extra context seemed to work really well. I also intuitively feel that when work is done in a sequential format, the AI seem to work pretty well. In the predictive task, for the final RandomForest code it picked up modifications from the previous cell and made the necessary adjustments.[sic]”* Regarding data science steps, some participants expressed positive views, with one stating *“Very positive experience. I believe the data science step can be very helpful when developing a project.”* However, two participants mentioned challenges in selecting the right step. This difficulty may arise from participants’ individual interpretation of the data science steps, or the complexity of choosing a single step when they may have wanted to combine multiple steps in one cell.

Continuing with the participants’ feedback, an ideal user interface should be more conversational. Three participants mentioned inline recommendations that follow a streaming nature, similar to existing assistants, would be nicer. However, they wrote *CATSci* was good for complex recommendations. One participant wrote *“Inline suggestions for small tasks A sidebar with tools for more complicated prompts (you did this very well!) A chat window for more casual exploration of features/functions.[sic]”*. Other mentions include further features for prompt engineering, formatting, and integrating the interface seamlessly with the IDE.

## Implications: Intelligent Assistant, Interaction and the Interface for Data Science Workflows

In this section, we discuss the insights, challenges, and opportunities revealed by the results (Sections 6 and 7) of our empirical study.

### Intelligent (AI) Assistant

#### Code Recommendations for Data Science

Our results show that recommendations are, in general, acceptable and useful, and there is a significant difference between recommendations provided for the descriptive task and the predictive task. Recommendations for predictive tasks were better in terms of both acceptance and performance. On the acceptability of a code recommendation, we suspect one of the major reasons could be due to the virtue of following similar, repetitive templates in predictive tasks. For example, lines of code written for a random forest classifier would follow a generic template set out by the corresponding library like Scikit except for elements such as variable names and hyperparameters and can be well mimicked by an LLM. In contrast, each descriptive data science task can be very unique in its nature and the state-of-the-art model is still limited in its ability to assist users in solving them. Our study highlights a significant challenge in the model’s capacity to meet user expectations for acceptable recommendations and its limitations in addressing descriptive data science tasks, even with repeated user requests. Additionally, assistants should be optimised for coding styles and conventions suitable for data science.

#### Alternatives in Data Science

Our analysis shows that having alternatives helps in the acceptance of recommendations, although not statistically significant. Our own direct probing of the utility of alternatives with the participants after the study showed that the alternative code recommendations revealed new libraries and syntax patterns, which participants found exciting. This is in line with McNutt et al. (2023)’s interview study that alternatives helped learn new patterns. A total of 9% of alternative recommendations were accepted in groups with alternatives, and two participants felt better alternatives were necessary to improve their usage. This shows that there is still considerable room for improving alternative recommendations that allow the exploration of analysis paths in data science tasks. This finding can help explain why code assistants are primarily used for improving productivity (McNutt et al. 2023) and not yet seen as suitable for exploration as revealed in interview-based studies (McNutt et al. 2023). Improved models that can provide appropriate alternatives that aid exploration may improve the usage of coding assistants also as a tool for exploration.

#### Capabilities

Participants expect the AI assistant to provide a comprehensive set of capabilities, including debugging errors, installing missing libraries before recommending their usage, and adding extensive markdowns or explanations when necessary. This means that typical code-completion tools, package installers, etc., may fall short of expectations as stand-alone tools and hinder their adoption in practice. Users also expect their capabilities to be seamlessly integrated into IDEs.

An ideal AI assistant, according to participants’ comments in the survey, should provide **faster** responses, follow **notebook conventions**, and have comprehensive capabilities. Additionally, it should be **conversational** (with memory) and is **aware of the data science workflow**.

### Interaction

#### Implications for Prompt Engineering

While LLMs like GPT are expected to do well in natural language tasks, our results show that participants struggle to get the desired performance out of the state-of-the-art GPT-4 model. Participants’ comments on the descriptive task indicate that they had to write additional instructions to get the recommendations they wanted, indicating that transforming the task descriptions into fairly precise and effective prompts is an arduous task for humans, especially descriptive tasks. In contrast, the majority of the participants generally had no difficulties in getting the code recommendation they were looking for predictive tasks.

Our analysis of the interaction log showed that, across all groups, in the context of descriptive tasks, participants had to (re)write instructions several times more ($\approx 40\%$ more than predictive tasks) and in a detailed manner to get the recommendations they wanted across all the groups (refer to results in Table 7). However, this effort is (statistically) significantly reduced to $\approx 15\%$ when data science step information is included in the prompt, showing the potential of workflow information.

It is important to note that the challenges associated with ineffective prompts may be further aggravated for non-native English speakers, given that a majority of the LLMs are designed for the English language. One participant explicitly wrote support for multiple languages would be helpful. It should be noted that translating business requirements into machine learning tasks is heavily human-reliant (Vogelsang and Borg 2019; Nahar et al. 2022). Therefore, future assistants for data science tasks should provide supportive tools in this regard, and any research into this area would be helpful.

### Interface

#### Enhanced, Intuitive IDE

User Interface insights from using CATSci show the users appreciate the simple, easy-to-use features. Moreover, our survey analysis reveals that users may develop confirmation bias over time, shaping their preferences for specific features such as inline recommendations available in existing assistants. Such bias based on previous experiences has also been revealed in other studies (McNutt et al. 2023). Therefore, when introducing new features in the interface for data scientists, it is necessary to understand any existing biases as they may lead to features being overlooked and impact adoption. At the same time, when introducing novel capabilities such as in the case of presenting alternative recommendations, our survey analysis shows that both the presentation style and their effectiveness are important.

## Limitations and Threats to Validity

### Generalisability of Results

It is important to emphasise that the tasks used in this study were of a simple nature, such as finding correlation and performing binary classification. Therefore, the results must be generalised with care. Extending the study to a larger set of data science tasks with different levels of difficulty and a larger pool of participants would be beneficial. Also, evaluation of AI assistants other than GPT-based models will provide further valuable insights to improve recommendations. However, both the quantitative and the survey results, complemented with participants’ comments, provide insights that can inform algorithmic and interface design for effective AI assistance in data science.

### Interaction History

One of the challenges is memory-less interaction to generate recommendations due to the nature of API calls. The *CATSci* interface is designed to address this to an extent by allowing the participants to pass the previous cells flexibly. Also, when solving a new step in the task workflow, there may be less reliance on previous recommendations. Still, it would be helpful for the assistant to be aware of the entire interaction in a notebook or, better, interaction across the notebook for a given user or a team(s). For example, exploiting the feedback feature of *CATSci* could already alleviate this to some extent. Hence, a solution that provides comprehensive conversational capabilities in IDE may be welcomed by the data science community.

### Extending for More Developer Activities

In our study, we considered several actions to record participants’ interaction with the AI assistant. Recording additional developer activities such as copy-pasting of code instead of clicking the *insert* button and deleting the code recommendation after inserting it into the cell could be helpful. Further studies could explore eye tracking or a fine-grained study of interaction logs.

### Response of AI Assistants

Another issue that was a source of inconvenience to participants was two minor restrictions in interacting with the interface: the input format and avoiding the usage of the variable name “data”. To display the code recommendations in a standardised format in light of the sometimes unpredictable nature of the response format to API calls, we used a pre-defined prompt (which was a result of several iterations). However, we restricted the usage of the special character ‘"’ (i.e., double-quote) in the instruction to avoid any input issues and usage of the variable name ‘data’ since this was used to record the interaction log. Participants wanted these restrictions to be gone. Without the restrictions, we believe the usability score of the interface could well improve. Addressing this in a future study should be easily possible. Also, a robust processing of responses that is compatible with different response formats or enforcing a standardised response format across the AI assistants that facilitates plug-in-and-play will be beneficial.

## Conclusion

In this work, we provide a comprehensive study, employing an experimental approach, on how data scientists interact with and use the code recommendations generated by LLM-based AI assistants for different tasks. Our study shows that while AI assistants exhibit the potential to aid data scientists in their tasks and generally receive positive impressions, there exist several areas of improvement in order for them to provide effective assistance or serve as effective pair-programming partners.

Our study shows that while alternatives can help discover new methods or syntax templates that provide a different way of implementing a function, they are not yet fully leveraged to effectively explore the diverse paths in the *garden of forking paths*. Further investigations into varied approaches for generating different sets of alternatives and optimising their presentation could be beneficial. Our findings indicate that explicitly including the data science step as context in the prompt significantly enhances the acceptability of recommendations.

Additionally, our study uncovers significant task-related differences across descriptive and predictive tasks in the acceptability and performance of the code recommendations generated by the AI assistant. This underscores the necessity to address the distinctive challenges posed by the former.

Finally, we identify several areas of improvement in both AI-generated recommendations and the interface to interact with them. Addressing these aspects is crucial for the successful adoption of valuable features into development tools for data scientists using notebooks, ultimately enhancing developer productivity.

## Supplementary Information

Below is the link to the electronic supplementary material.

Supplementary file 1 (pdf 457 KB)

## Data Availability

The datasets generated and analysed in this study are accessible in anonymised form as part of our replication package at the following link: https://doi.org/10.5281/zenodo.10943330. The dataset and task files used in the experiment are openly accessible at https://doi.org/10.5281/zenodo.13639707.

## Footnotes

1 chained as [cell_1 + .. + cell_n] where cell_1 is identified as the previous cell that appears immediately before the cell asking recommendation

2 https://www.cdc.gov/nchs/covid19/pulse/mental-health.htm

3 https://www.kaggle.com/datasets/mlg-ulb/creditcardfraud/data

## Code Availability

All supporting data and materials for the findings of this study can be accessed in our replication package: https://doi.org/10.5281/zenodo.10943330.
