# Supplementary material for: AI support for data scientists: An empirical study on workflow and alternative code recommendations
Source: Empir Softw Eng. 2025 Jul 4;30(5):133. doi: 10.1007/s10664-025-10622-4 (PMC12227384; doi:10.1007/s10664-025-10622-4)
Supplement: Supplementary file 1 — (pdf 457 KB) [file 10664_2025_10622_MOESM1_ESM.pdf]

Experiment Instructions

(Note: The following instructions correspond to *Alt.DS* group, which includes all experimental conditions. They serve as a representative reference, as other groups received similar instructions with minor condition-specific variations based on their assigned conditions.)

Consent Form

Welcome to the experiment.

This experiment aims to understand the effectiveness of code recommendations provided by AI assistants for solving data science tasks. That is, how helpful they are in accomplishing the tasks without extensive manual coding.
Additionally, the experiment explores if the interface to communicate with the AI assistant within the Jupyter notebook environment provided is helpful.

The AI assistant throughout this experiment is GPT-4.
You should use the Chrome or Safari browser for this experiment and use a desktop or laptop as the device.
Please keep this survey window always open. Open any new links provided in this experiment in a new tab.

We request you read the instructions carefully and follow them throughout this experiment.

The experiment consists of four parts (Part 1-3).
In Part 1, you will answer a set of questions about your experience with AI assistants.
In Part 2, you will get detailed information about the Jupyter environment and will be given two (one descriptive and one predictive) data science tasks to solve and answer a set of questions.
In Part 3, you will answer a set of questions about your experience in Part 2.

In this experiment, you will receive:

- a survey questionnaire to record your experience and solutions
- two data science tasks to solve and corresponding data files
- a notebook (Jupyter) environment to solve the tasks with access to GPT through a custom interface

During the experiment, we will collect the following data:
1. within the Jupyter environment, we collect the log data of interaction with the AI assistant
- any query sent to GPT API
- recommendation responses received from GPT API
- actions taken on the recommendation (accept and insert, reject, edit)
- feedback provided for the recommendation
- copies of the .ipynb notebook (every minute)

2. solution notebooks for the tasks
3. response collected through a questionnaire

The experiment should take a maximum of 2 hours in total. Make sure you are in an environment where you are not distracted or interrupted by other tasks or things during the entire duration of this experiment.

Compensation: You will receive a standard payment of 30$ for completing the experiment, plus 5$ for your feedback, plus an opportunity to earn an additional payment of upto 10$ by solving the data science tasks correctly.
(By `completing the experiment`, we mean following the instructions carefully and sufficiently interacting with the AI assistant to finish the tasks. The environment provided should only be used to complete the given tasks.)

The payment will be made to your account within 10 days of completing the experiment.

Data usage: All the data collected during the entire process of this experiment will be anonymized and used for research and documentation purposes only. The queries to GPT and the responses from GPT follow data guidelines set by OpenAI. However, the API calls will be associated with a separate ID for this experiment and, therefore, are not linked to you personally.

By taking part in this experiment,

- ○ I consent to the conditions and usage of my anonymized data for research purposes.
- ○ I do not consent.

Profile ID

What is your profile ID?
(please indicate your public profile id in the platform)

Main Survey - Part 1 Questionnaire

Part 1 - Questions about your experience

Do you use AI assistants (e.g., ChatGPT, Co-Pilot) for **programming tasks**?

- ○ Never
- ○ Rarely (less than 4 times in a month)
- ○ Sometimes (1-2 times in a week)
- ○ Very Often (more than 3 times in a week)
- ○ Always (in day-to-day tasks)

If you use AI assistants for **programming tasks**, indicate the name(s) of the assistant(s).

Do you use AI assistants (e.g., ChatGPT, Co-Pilot) for **data science programming tasks**?

- ○ Never
- ○ Rarely (less than 4 times in a month)
- ○ Sometimes (1-2 times in a week)
- ○ Very Often (more than 3 times in a week)
- ○ Always (in day-to-day tasks)

If you use AI assistants for **data science programming tasks**, indicate the name(s) of the assistant(s).

Do you use AI assistants in **Jupyter Notebooks**?

- ○ Never
- ○ Rarely (less than 4 times in a month)
- ○ Sometimes (1-2 times in a week)
- ○ Very Often (more than 3 times in a week)
- ○ Always (in day-to-day tasks)

If you use AI assistants in **Jupyter Notebooks**, indicate the name(s) of the assistant(s).

Main Survey - Part 2 Data science tasks

Part 2 - Solving Data Science Tasks

To solve the data science tasks, please use the Jupyter environment that we will provide you and Python as the programming language.

Within the Jupyter environment, you will have access to an AI assistant. This assistant generates the recommendations for the next code block with the click of a button. **This setup allows you to get code recommendations without leaving your development environment.**
You can use the AI-generated code recommendations to solve the tasks.

Since we want to understand your experience interacting with an AI assistant within the Jupyter Notebook environment, **we request you use the AI assistant as much as possible to finish your tasks without writing the code from scratch yourself.**

Please select strongly agree to show that you are paying attention to this question.

- ○ Strongly disagree
- ○ Somewhat disagree
- ○ Neither agree nor disagree
- ○ Somewhat agree
- ○ Strongly agree

Interacting with the AI assistant inside Jupyter

As you already know, the AI assistant in this experiment is GPT-4.
The API call to GPT-4 considers each request a standalone one. That is, it provides a memory-less interaction. For each request you send, it provides a response.

In the Jupyter notebook environment that you will receive, you can send the API request with the click of a button (see image below) without having to write a code-based API call or prompt yourself.

Get new recommendation for cell *with alternatives*

To send the request, you have to select the

1. data science step (*mandatory) - the data science step you want the recommendation for.

A predefined set of data science steps is provided to choose from with explanations. You can find the description of what each data science step means by hovering over the step in the interface.

Additionally, you can select several information in order to provide more context to each request.
The context information you can select includes (see image below):

2. previous cells (*optional) - includes the content in the number of previous cells selected (by default, all the previous cells are selected)
3. additional instructions (*optional) - additional instruction text you want to add (e.g. load titanic dataset)

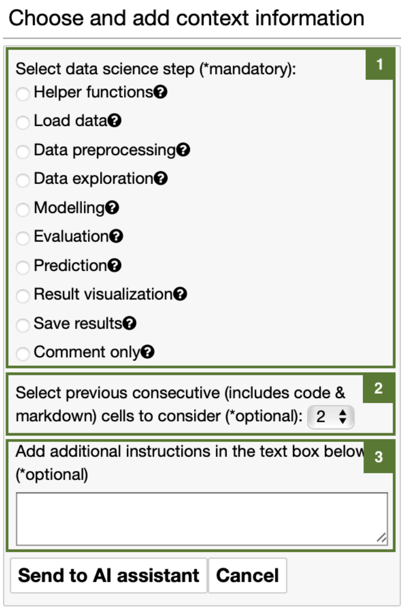

Once you select all the context information and click 'Send to AI assistant', we will send a request to the AI assistant to get the recommendation for the next code block.
This request to the AI assistant contains a pre-defined prompt query template that is filled based on the context information you selected.

The response for each request is a code block recommendation, along with the top three alternatives and an explanation.
Please note that it will take a few to several seconds to receive the response from API.

Please click the button below to see an example response.

An example response is shown below:

response from AI assistant:

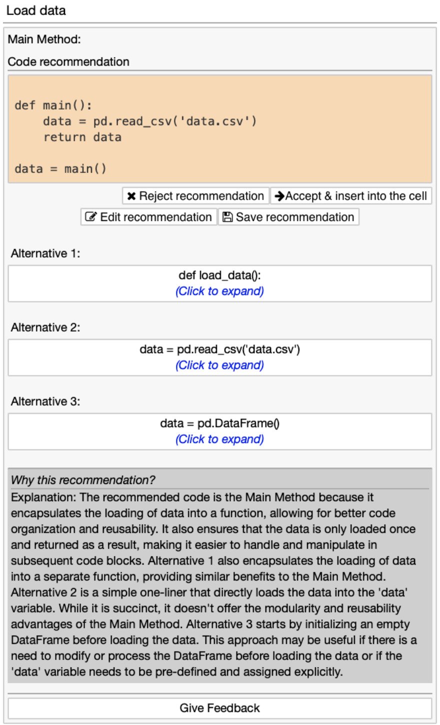

You can reject, accept, edit, and save the recommendation using the buttons provided.

For each recommendation provided, you can provide feedback by clicking the *Give Feedback* button shown below.

Give Feedback

Before you do the tasks, please follow the next steps to familiarise yourself with the experiment setup.

Step 1. Please go through the introduction video showcasing the notebook environment you will receive.

Please pay attention to the video as the setup and all its features are explained to you in the video.

You can find the video in the link: https://youtu.be/-xQkgvsI8Rw

Once you have gone through the video, please click the button below to proceed.

Please ask the experimenter to send the link to the Jupyter notebook setup.

Once you receive the link, please click the button below to proceed.

Step 2: Now, please take the next five minutes to try the setup yourself before the task starts.

You can do this in the test notebook 'test.ipynb' available in the test folder in the link you received.

If you face any issues, try refreshing the page. If the issue persists, let the experimenter know.

Once you are done with testing the setup using 'test.ipynb', please click the button below to proceed.

(please do not close this window in the meantime)

Solving data science tasks

Now that you are familiar with the setup, we ask you to solve two data science tasks, one descriptive task* and one predictive task**, in the same order in which it is provided to you.
Each task is expected to take approximately 15 minutes. Once you finish the first task, proceed to the second task.

Please make sure you:

- **use the setup** provided.
- **interact with the AI assistant** to do your task instead of writing the code from scratch yourself.
- **provide feedback on each recommendation** you receive from the AI assistant.
- please do not use 'data' as a variable name in your solutions since it is predefined for other purposes in this experiment. Please avoid using the character " in additional instructions if you encounter any issues obtaining a recommendation.

**Descriptive task: Descriptive tasks summarise, explore, or visualise the data that is collected in order to, for example, find patterns.*
***Predictive task: Predictive tasks use methods like learning algorithms to infer and make predictions on future data.*

Note that for each task, you can earn upto 5$ for solving the tasks correctly (as explained more in each of the task description).

Please click the button below to do the first task.

Descriptive task

The household Pulse Survey was conducted by the National Center for Health Statistics (NCHS) together with the Census Bureau in order to monitor changes in mental health over certain indicators over time. The survey was conducted over several weeks across the states of the USA in order to provide information about the impact of the COVID-19 pandemic. The data and information is published at https://www.cdc.gov/nchs/covid19/pulse/mental-health.htm

The data file required for this task is:
'data/Processed_Indicators_of_Anxiety_or_Depression_Based_on_Reported_Frequency_of_Symptoms_Over_Last_Two_Weeks.csv' (available in the data folder that we provide you).

The data available to you is already preprocessed and contains indicators measured over the period of July 21, 2021 - Aug 7, 2023.

The indicators in the processed data are based on the collected information on the self-reported frequency of anxiety and depression symptoms over the last two weeks from the measurement day.

It contains the following information:
- **Indicator**: symptom indicator
- Group: denotes the measurement group
- **State**: provides the state information
- Subgroup: provides the state subgroup
- Phase: provides the phase information of the study
- Time Period: provides the time period for the measurement
- Time Period Start Date: provides the start date of the time period for the measurement
- Time Period End Date: provides the end date of the time period for the measurement
- **Value**: The value provides the percentage of adults aged 18 and over having symptoms of anxiety disorder and symptoms of depressive disorder and symptoms of anxiety disorder or depressive disorder.
- The data also contains the lower value of the confidence interval (Low CI), the higher value of the confidence interval (High CI), the Confidence Interval, and the Quartile Range.

Having extracted the data from 51 states for 27 collection periods, we now want to run some analysis.

Let us select the top three highly populated states of US (California, Texas, Florida) and create three pairs out of them.
Pair 1: California, Texas
Pair 2: Texas, Florida
Pair 3: Florida, California
As you know, order does not matter in finding correlation between each of the above pairs.

Now, your task is to find the pair which shows highest correlation w.r.t the 'values' of the indicator 'Symptoms of Anxiety Disorder' among the above three pairs.

Hint: For each pair, filter the values based on specified Indicator and State column. Then, find correlation between the values.

If you find the top pair correctly, you will get a bonus payment of 2.5$.
If you find the correlation value correctly, you will get a bonus payment of 2.5$.
So, if you answer both top pair and correlation value correctly, you will get a bonus payment of 5$.

In order to do the task, open the notebook descriptive.ipynb in the descriptive folder.

After you finish the task, please click the button below to proceed.

(please do not close this window in the meantime)

Did you finish the descriptive task?

- ○ Yes
- ○ No

Please enter your solution to the descriptive task

1. Please enter the top pair of states in the format: state1, state2 (states in no particular order).	
2. Please enter the correlation value of the top pair of states. (rounded to two decimals)	

Please rate your confidence in your solution to the descriptive task

- ○ Not confident at all
- ○ Slightly confident
- ○ Somewhat confident
- ○ Fairly confident
- ○ Completely confident

I believe my solution to the descriptive task (select all applicable)

	Yes	No
has interpretable methods	○	○
is understandable for someone without my help	○	○

**Interpretable methods are those who internal workings, that is, how it makes decisions, can be understood by humans. For example, simple decision tree models are easy for humans to interpret, whereas, deep learning methods are generally less interpretable.*

Please click the button below to proceed.

Predictive task

In this task, you will receive two datasets that capture various features of financial data that can belong to a 'Class' (0 or 1).

One dataset is titled train.csv, and the other is titled test.csv (available in the data folder that we provide you ‘data/train.csv’ and ‘data/test.csv’). Due to the confidential nature of the data, the data presented to you contains already preprocessed features.

Train.csv and Test.csv have both the same set of features. Additionally, train.csv has 'Class' information to denote whether it belongs to the 'Class' or not. This is marked by experts and is the 'ground truth'.

The test.csv dataset does not reveal the ground truth. **Your task is to predict this based on the patterns in the training dataset.**

Once your predictions for test.csv are ready, save them as 'results.csv' with column name ['class'] in the data folder ('data/results.csv') for evaluation by the experimenter.

If your predictions achieve an accuracy of >=99%, you will get a bonus payment of 5$.
If your predictions achieve an accuracy of 90% to <99%, you will get a bonus payment of 2.5$.

In order to do the task, open the notebook *predictive.ipynb* in the predictive folder.

After you finish the task, please click the button below to proceed.

(please do not close this window in the meantime)

Did you finish the predictive task?

- ○ Yes
- ○ No

Please enter your solution (name of the ML model) to the predictive task

[ ]

Did you save your results.csv file in the folder?

- ○ Yes
- ○ No

Please rate your confidence in your solution to the predictive task

- ○ Not confident at all
- ○ Slightly confident
- ○ Somewhat confident
- ○ Fairly confident
- ○ Completely confident

I believe my solution to the predictive task (select all applicable)

	Yes	No
has interpretable methods	○	○
is understandable for someone without my help	○	○

**Interpretable methods are those who internal workings, that is, how it makes decisions, can be understood by humans. For example, simple decision tree models are easy for humans to interpret, whereas, deep learning methods are generally less interpretable.*

Please click the button below to proceed.

Finished tasks

You have now completed both the data science tasks. During the experiment, you had access to an AI assistant (ChatGPT) for recommending the next(-step) code block in the notebook workflow.

Please click the button below to proceed to the final part of this experiment.

Main Survey - Part 3 Questionnaire

Part 3 - Questions about your experience in the tasks

Through the following set of questions, we would now like to understand your impression and experience with using the AI assistant for solving data science tasks.

There are two subparts in Part 3.
1. Questions about the recommendations
2. Questions on the interface

Please take time to read each question carefully and provide your answer.

1A. Requesting the recommendation

1. For each request, I would prefer to receive recommendation

○ with alternatives.
○ without alternatives.

2. With each request, I prefer to control the next step of the workflow by specifying the data science step I need recommendations for

○ Yes
○ No

3. With each request, I prefer to send the following information as the context (select all applicable)

☐ data science step
☐ previous cells
☐ additional instructions

4. I would rely on the AI assistant for the step

	Never	Sometimes	About half the time	Most of the time	Always
	0	20	40	60	80	100
Helper functions					
Load data					
Data preprocessing					
Data exploration					
Modelling					
Evaluation					
Prediction					
Result visualization					
Save results					
Comment only					

1B. Understanding the recommendation

1. I can understand the recommended code without much difficulty.

- Yes
- No

Please use the text box below to elaborate on your answer to the above question.

2a. Please rank in order of importance, from highest to lowest, the context information below you think **were instrumental in getting the response you wanted**.
(drag to order)

data science step

previous cells

additional instructions

2b. Please rank in order of importance, from highest to lowest, the context information below you think **were instrumental in getting the response you found helpful**.
(drag to order)

data science step

previous cells

additional instructions

3a. Pre-defined prompts

	The pre-defined prompts **were sufficient to get the response I wanted.**	The pre-defined prompts **were not sufficient**, and I had to write further instructions **to get the response I wanted**.
Select the applicable statement. Please use the text box below to elaborate.	○	○

3b. Pre-defined prompts

	The pre-defined prompts were **sufficient to get the response I found helpful.**	The pre-defined prompts **were not sufficient**, and I had to write further instructions **to get the response I found helpful**.
Select the applicable statement. Please use the text box below to elaborate.	○	○

(For all the questions, please use the text field provided to elaborate on your answer.)

1C. Evaluating the recommendation

1. Expectation

	in terms of content	in terms of quality
The first recommendations are as I expected.	☐	☐
The alternatives are as I expected.	☐	☐

2. Utility

	Yes	No
The recommendations with alternatives are helpful in solving the tasks.	○	○
The recommendations with alternatives are helpful in exploring new paths in my analysis.	○	○
I gained new knowledge from the recommendations with alternatives.	○	○

(For all the questions, please use the text field provided to elaborate on your answer.)

1D. Using the recommendation (including the alternatives)

	Strongly disagree	Somewhat disagree	Neither agree nor disagree	Somewhat agree	Strongly agree
I can use the recommended code without much difficulty.	○	○	○	○	○
I think I would have taken longer to complete the task without the recommendations.	○	○	○	○	○
The recommendations were helpful in exploring different methods.	○	○	○	○	○
The recommendations were helpful in doing a thorough analysis.	○	○	○	○	○
I would use the AI assistant (again) for data science tasks.	○	○	○	○	○

(For all the questions, please use the text field provided to elaborate on your answer.)

1E. Recommendations for descriptive task vs. predictive task

	descriptive task	predictive task	equally both tasks
The first recommendations were more helpful for			
The alternative recommendations were more helpful for			

1F. Please elaborate on your task-related experiences (both positive and negative), challenges encountered, and any workarounds you attempted regarding the recommendations.

1G. Please elaborate on your expectations and needs for the ideal solution regarding the recommendations.

2A. Feedback on the interface to interact with the AI assistant

	Strongly disagree	Somewhat disagree	Neither agree nor disagree	Somewhat agree	Strongly agree
I find the interface helpful to interact with the AI assistant.					
I find the cell-based recommendations intuitive to use.					
The interface contains all the functionalities that I needed during the experiment.					
I would like to use this interface again.					

2B. Usability of the interface

	Strongly disagree	Somewhat disagree	Neither agree nor disagree	Somewhat agree	Strongly agree
I think that I would like to use this interface for data science tasks frequently.	○	○	○	○	○
I found the interface unnecessarily complex.	○	○	○	○	○
I thought the interface was easy to use.	○	○	○	○	○
I think that I would need the support of a technical person to be able to use this interface.	○	○	○	○	○
I found the various functions in this interface were well integrated.	○	○	○	○	○
I thought there was too much inconsistency in this interface.	○	○	○	○	○
I would imagine that most people would learn to use this interface very quickly.	○	○	○	○	○
I found the interface very cumbersome to use.	○	○	○	○	○
I felt very confident using the interface.	○	○	○	○	○
I needed to learn a lot of things before I could get going with this interface.	○	○	○	○	○

2C. Please elaborate on your experiences (both positive and negative) and challenges encountered with respect to the interface to interact with the AI assistant for data science programming.

2D. Please elaborate on your expectations or needs for the ideal interface to interact with the AI assistant for data science programming.

End of Survey message

You have now completed the experiment. We thank you very much for your valuable participation.

We will be in touch with you soon regarding the payment. Please log out of the notebook environment and proceed to register your submission.

Powered by Qualtrics
